# Multimodal Phase-Space Dynamics Fusion for Robust Ischemia Screening: An Edge-AI Paradigm with SERF Magnetocardiography

**DOI:** 10.3390/bios16040228

**Published:** 2026-04-20

**Authors:** Keyi Li, Xiangyang Zhou, Yifan Jia, Ruizhe Wang, Yidi Cao, Jiaojiao Pang, Rui Shang, Yadan Zhang, Yangyang Cui, Dong Xu, Min Xiang

**Affiliations:** 1 Key Laboratory of Ultra-Weak Magnetic Field Measurement Technology, Ministry of Education, School of Instrumentation and Optoelectronic Engineering, Beihang University, Beijing 100191, China; likeyi@buaa.edu.cn (K.L.); xyzhou@buaa.edu.cn (X.Z.); jyf@buaa.edu.cn (Y.J.); wangruizhe@buaa.edu.cn (R.W.); caoyidi_buaa@buaa.edu.cn (Y.C.); 2 Zhejiang Provincial Key Laboratory of Ultra-Weak Magnetic-Field Space and Applied Technology, Hangzhou Innovation Institute, Beihang University, Hangzhou 310051, China; 3 Hefei National Laboratory, Hefei 230088, China; 4 Department of Emergency Medicine, Qilu Hospital of Shandong University, Jinan 250012, China; jiaojiaopang@email.sdu.edu.cn (J.P.); 18560083003@163.com (R.S.); 5 Shandong Key Laboratory for Magnetic Field-Free Medicine & Functional Imaging, Qilu Hospital of Shandong University, Jinan 250012, China; 6 Shandong Provincial Clinical Research Center for Emergency and Critical Care Medicine, Qilu Hospital of Shandong University, Jinan 250012, China; 7 NMPA Key Laboratory for Clinical Research and Evaluation of Innovative Drug, Qilu Hospital of Shandong University, Jinan 250012, China; 8 Hangzhou Institute of National Extremely-Weak Magnetic Field Infrastructure, Hangzhou 310028, China; zhang-yd20@tsinghua.org.cn; 9 State Key Laboratory of Traditional Chinese Medicine Syndrome, National Institute of Extremely-Weak Magnetic Field Infrastructure, Hangzhou 310028, China

**Keywords:** SERF magnetocardiography, Edge AI, phase-space reconstruction, deep learning, ischemia screening, Portable Biosensors

## Abstract

Background: Myocardial ischemia (MI) is a major cause of morbidity and mortality worldwide and requires timely and reliable detection. Although Spin-Exchange Relaxation-Free (SERF) magnetocardiography (MCG) provides femtotesla-level sensitivity for identifying non-linear cardiac repolarization anomalies, its clinical deployment is currently impeded by the computational bottlenecks inherent to portable edge platforms. Methods: We propose a “Sensor-to-Image” Edge-AI framework that links quantum sensing with computer vision. Single-channel SERF-MCG signals from a large cohort of 2118 subjects (1135 Healthy, 983 Ischemia) were transformed into phase-space images using three distinct encoding modalities: Recurrence Plots (RP), Gramian Angular Summation Fields (GASF), and Markov Transition Fields (MTF). These visual representations were subsequently analyzed by a streamlined MobileNetV3-Small architecture, optimized for low-latency inference. To maximize diagnostic precision, an adaptive weighted fusion mechanism was engineered to combine the chaotic specificity captured by RP with the morphological sensitivity of GASF through a validation-optimized fixed global weighting strategy. Results: In our experiments, the fusion model achieved an Area Under the Curve (AUC) of 0.865, which was higher than the 1D-CNN baseline (AUC 0.857) and the single-modality models. Notably, the fusion strategy significantly elevated sensitivity to 88.3% while maintaining a specificity of 66.5%. Although specificity is moderate, this trade-off prioritizes high sensitivity to minimize false negatives in pre-hospital screening scenarios. The average inference time was 4.7 ms per sample on a standard CPU, suggesting suitability for real-time Point-of-Care (PoC) scenarios under further on-device validation. Conclusions: The results suggest that multi-view phase-space fusion can capture subtle spatio-temporal changes associated with ischemia. The proposed lightweight framework may support the development of portable SERF-MCG systems with embedded AI screening.

## 1. Introduction

Cardiovascular diseases (CVDs) persist as the leading cause of mortality worldwide [1]. Myocardial ischemia, a common precursor to infarction, often causes subtle electrophysiological changes that may not be detected by standard 12-lead electrocardiography (ECG), especially in non-ST-elevation cases. This diagnostic limitation is largely attributed to the attenuation and distortion of electrical signals as they propagate through thoracic tissues. In contrast, Magnetocardiography (MCG) measures the magnetic fields generated by cardiac currents. According to the Biot-Savart law, magnetic fields permeate biological tissues with minimal distortion, theoretically offering superior sensitivity to local repolarization dispersion and tangential currents [2,3].

Historically, the clinical adoption of MCG has been constrained by the reliance on Superconducting Quantum Interference Devices (SQUIDs), which necessitate bulky cryogenic cooling [4]. Spin-Exchange Relaxation-Free (SERF) atomic magnetometers now provide ultra-high sensitivity (∼10 fT/$\sqrt{\mathrm{Hz}}$) at room temperature [5,6]. These sensors suggest the theoretical feasibility of portable magnetic cardiography using OPM/SERF technologies. However, as noted in the broader neuroimaging literature [7,8], practical wearable MCG deployment specifically for cardiac applications still faces significant engineering constraints, making edge-efficient algorithmic design a critical prerequisite for future bedside use.

However, analyzing SERF-MCG signals on portable devices introduces a practical trade-off between model accuracy and computational cost. Biological magnetic signals are inherently chaotic and non-stationary [9]. Deep learning models such as ResNet or Transformers can achieve high accuracy, but their computational requirements often make them difficult to deploy on battery-powered edge devices [10,11]. Conversely, shallow models often fail to capture the complex non-linear dynamics associated with ischemia.

**Figure 1:**
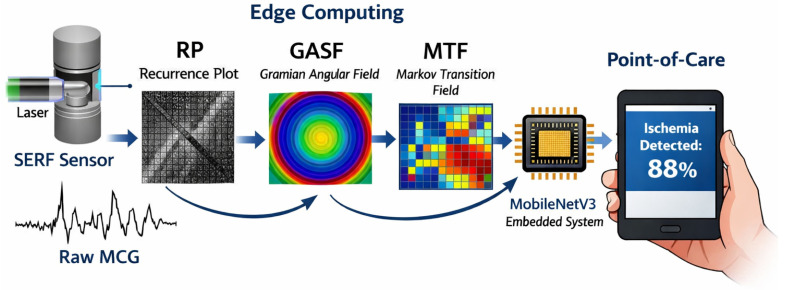
Schematic diagram of the proposed “Sensor-to-Image” edge-AI framework. High-sensitivity SERF-MCG signals are transformed into phase-space images and analyzed by a lightweight MobileNetV3 for real-time ischemia screening.

To address this issue, we propose a multimodal phase-space fusion framework (Figure 1). Rather than relying on direct 1D signal analysis, we project time-series data into 2D phase space using Recurrence Plots (RP), Gramian Angular Fields (GASF), and Markov Transition Fields (MTF), which provide complementary representations of non-linear temporal dynamics and recurrence structure [12,13]. These images serve as inputs for MobileNetV3 [14], a widely used lightweight convolutional neural network. By fusing the complementary features of chaos (extracted via RP) and morphology (extracted via GASF), our system achieves robust screening performance compatible with edge deployment constraints [15,16].

## 2. Related Work

The intersection of quantum sensing and artificial intelligence has spurred significant interest in automated MCG analysis. While SQUID-MCG has been extensively studied, SERF-MCG remains a nascent field. Steinisch et al. [17] proposed an automatic classification framework for coronary artery disease based on entropy-related features extracted from MCG recordings; however, such feature-engineering-based approaches may be difficult to translate to simplified portable settings. Convolutional neural networks (CNNs) are widely used in biomedical time-series analysis, and deep learning models have shown strong diagnostic performance in ECG-related tasks [18], although standard deep architectures often remain computationally demanding in resource-constrained settings.

In the domain of time-series classification, transforming 1D signals into 2D images has gained traction as a method to leverage computer vision models. Wang and Oates [12] proposed a framework for encoding time series as images, including Gramian Angular Fields and Markov Transition Fields, thereby enabling image-based classification of temporal data. Marwan et al. [13] reviewed Recurrence Plots as a powerful tool for visualizing and analyzing complex dynamical systems. However, most existing studies utilize heavy backbones like ResNet-50 or Transformers, which are ill-suited for resource-constrained edge devices. While Acharya et al. [19] explored deep convolutional neural networks for ECG-based myocardial infarction detection, their application to the more complex, non-stationary SERF-MCG signals remains unexplored. Our work addresses this gap by synergizing phase-space encoding with an edge-optimized neural architecture.

## 3. Materials and Methods

### 3.1. Study Population and SERF-MCG Instrumentation

We retrospectively analyzed SERF-MCG recordings from 2118 participants recruited at Qilu Hospital of Shandong University. The study protocol adhered to the Declaration of Helsinki and was authorized by the local Institutional Review Board (No. KYLL-202204-017). Informed consent was obtained from all subjects. As a single-center retrospective study, the findings should be interpreted with caution regarding broader population generalizability.

To mitigate the influence of major confounding factors on cardiac repolarization, highly stringent inclusion and exclusion criteria were established for the Healthy Control (HC) group (n = 1135), informed by the Chinese health industry standard WS/T 402-2012 [20] and routine clinical screening practice. Participants were required to be ≥18 years old with no hospitalizations in the preceding six months. Exclusion criteria for the HC group explicitly eliminated conditions known to alter electrophysiological features, including: (1) any history of cardiovascular diseases (e.g., coronary heart disease, structural heart disease, arrhythmias, heart failure); (2) cardiovascular risk factors including hypertension, diabetes mellitus, and hyperlipidemia; (3) current use of prescription medications; (4) abnormal vital signs, defined as body mass index (BMI) outside 18–28 kg/m^2^, resting heart rate outside 50–110 bpm, or blood pressure ≥140/90 mmHg; (5) abnormal fasting blood glucose (≥7.0 mmol/L) or total cholesterol (≥6.2 mmol/L); and (6) any pathological findings on 12-lead ECG (e.g., ST-T changes, arrhythmias) or echocardiography (e.g., left ventricular hypertrophy and valvular regurgitation).

The Myocardial Ischemia (MI) group (n = 983) comprised a real-world retrospective clinical cohort encompassing diverse clinical pathways, including both acute presentations and long-term follow-ups. Consequently, a uniform time interval between the SERF-MCG acquisition and specific reference tests (such as CAG) varied among individuals, and not all patients underwent invasive angiography. Diagnoses were definitively established by experienced cardiologists based on the patients’ comprehensive clinical records at the time of the MCG scan, integrating symptoms, 12-lead ECG, and available concurrent or historical reference tests (CAG or stress echocardiography). Patients with pre-existing arrhythmias or pacemakers were explicitly excluded to isolate ischemia-driven repolarization heterogeneity.

The magnetic signals were acquired using a custom-built 36-channel atomic magnetometer array based on SERF technology (Beihang University). The sensors operate with ^87^Rb vapor cells maintained at 150 °C, achieving an ultra-high sensitivity of ∼10 fT/$\sqrt{\mathrm{Hz}}$ (0–100 Hz bandwidth) inside a magnetically shielded room [5]. To emulate a single-probe portable operational scenario under limited edge-computing resources, we utilized the signal from a single representative sensor (Channel 5). It is important to note that this channel was selected arbitrarily prior to model training, rather than being retrospectively chosen as the central or “best-case” channel. By deliberately avoiding performance-based cherry-picking, the reported metrics serve as a conservative baseline for single-channel edge-AI feasibility. We acknowledge that in a real-world clinical setting, blindly stabilizing a single probe is practically challenging. As discussed in Section 5.4, consistent with our prior methodologies, our actual translational objective relies on a sparse array (e.g., 4–9 channels) to perform spatial signal averaging, which naturally circumvents precise anatomical placement requirements and further enhances the signal-to-noise ratio.

### 3.2. Signal Preprocessing and Segmentation

Raw SERF-MCG signals inevitably contain environmental noise, including 50 Hz power-line interference and baseline drift caused by subject movement. We applied a fourth-order Butterworth bandpass filter (0.5–100 Hz) followed by a 50 Hz notch filter. To ensure consistency across phase-space reconstructions, Z-score normalization was applied to each recording:

(1) $${\tilde{x}}_{i}=\frac{x_{i}-\mu }{\sigma }$$

where $x_{i}$ denotes the original signal amplitude at the *i*th sampling point, μ and σ represent the mean and standard deviation of the recording, respectively, and ${\tilde{x}}_{i}$ denotes the normalized signal. Z-score normalization was used for amplitude stabilization at the recording level, while Min–Max normalization was used as the direct input to time-to-image encoders. For time-to-image encoding (RP/GASF/MTF), each segmented cycle was mapped to the interval [0,1] using Min–Max scaling. This normalized signal $x_{i}\in \left[0,1\right]$ was directly used as the input for all subsequent image encoders. The continuous recordings were segmented into individual cardiac cycles using a modified Pan-Tompkins algorithm adapted for magnetic signals [21]. Each segment was resampled to a fixed length of N=224 points to align with the input requirements of the neural network. To prevent information leakage across temporally adjacent segments from the same subject, the dataset was split at the subject level into training (70%), validation (10%), and test (20%) sets. Stratified sampling was applied to preserve the class ratio across splits. It should be clarified that while the data splitting was strictly performed at the subject level to prevent data leakage, the final diagnostic metrics reported in this study were evaluated at the cycle level. Each individual segmented cardiac cycle was classified independently without multi-cycle aggregation per patient, aiming to rigorously assess the model’s sensitivity to instantaneous, single-beat repolarization anomalies.

### 3.3. Phase-Space Imaging Encodings

We implemented three distinct encoding methods to capture the multifaceted dynamics of the cardiac magnetic field.

#### 3.3.1. Recurrence Plot (RP): Visualizing Chaos

**Figure 2:**
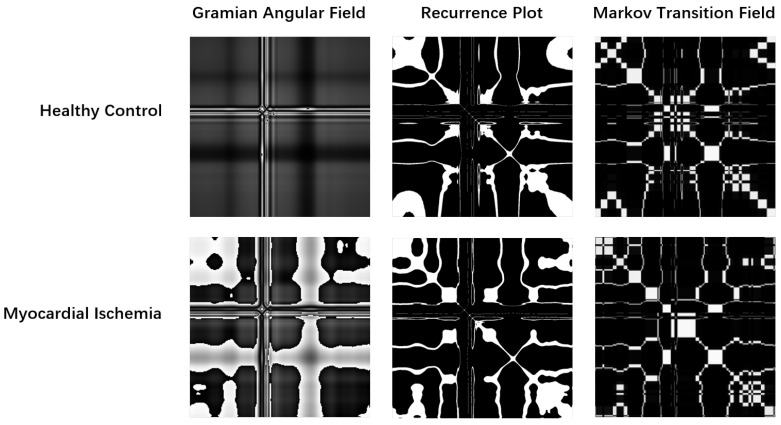
Visual comparison of phase-space encoding strategies. The top row (Healthy Control) exhibits regular, symmetric textures and clear grid-like structures. The bottom row (Myocardial Ischemia) reveals chaotic disruptions, high-frequency ripples, and fragmented trajectories.

RP analysis is grounded in chaos theory and provides a visualization of the recurrence of states in phase space [13]. Following Takens’ embedding theorem [22], the phase space trajectory ${\vec{s}}_{i}\in R^{m}$ is reconstructed from the univariate time series via time delay embedding:

(2) $${\vec{s}}_{i}=\left(x_{i},x_{i+\tau },\ldots ,x_{i+(m-1)\tau }\right)$$

where *m* is the embedding dimension and τ is the time delay. Optimal parameters (m=3,τ=4) were determined using the False Nearest Neighbors (FNN) method [23] and Mutual Information analysis [24]. The RP matrix $R_{i,j}$ is defined as:

(3) $$R_{i,j}=\Theta (\epsilon -||{\vec{s}}_{i}-{\vec{s}}_{j}{\left|\right|}_{2}),\;i,j=1,\ldots ,N$$

where Θ is the Heaviside step function and ϵ is a threshold distance. As illustrated in Figure 2, healthy signals typically exhibit regular, grid-like textures, whereas ischemic signals display disrupted, chaotic patterns indicative of repolarization instability [9].

#### 3.3.2. Gramian Angular Field (GASF): Encoding Morphology

GASF encodes temporal correlations in a polar coordinate system, thereby preserving the temporal dependency of the signal [12]. To ensure mathematical validity of the inverse cosine mapping, the Min–Max normalized signal $x_{i}\in \left[0,1\right]$ was linearly mapped to ${\bar{x}}_{i}\in \left[-1,1\right]$ prior to angular encoding:

(4) $${\bar{x}}_{i}=2x_{i}-1$$

The rescaled time series is then mapped into the angular domain for GASF construction:

(5) $$\phi_{i}=\arccos\left({\bar{x}}_{i}\right)$$

The GASF matrix is computed as the trigonometric sum:

(6) $$G_{i,j}=\cos\left(\phi_{i}+\phi_{j}\right)={\bar{x}}_{i}{\bar{x}}_{j}-\sqrt{1-{\bar{x}}_{i}^{2}}\sqrt{1-{\bar{x}}_{j}^{2}}$$

This formulation yields a structured 2D representation that preserves temporal dependency and salient morphological characteristics (e.g., QRS width and ST-segment slope) for downstream convolutional learning.

#### 3.3.3. Markov Transition Field (MTF): Statistical Transitions

MTF captures the transition probabilities among discretized states of the time series and projects the Markov dynamics onto an N×N field [12]. Specifically, the normalized sequence is first discretized into *Q* quantile bins (we set Q=8 in this study), yielding a state assignment function q(·) that maps each sample to its quantile index. A first-order Markov transition matrix $P\in R^{Q\times Q}$ is then estimated as:

(7) $$P_{a,b}=P\left(q\left(x_{t+1}\right)=b|q\left(x_{t}\right)=a\right),\;a,b\in \left\{1,\ldots ,Q\right\}.$$

Finally, the Markov Transition Field is constructed by aligning the transition probabilities with temporal indices:

(8) $${\mathrm{MTF}}_{i,j}=P_{q\left(x_{i}\right),\;q\left(x_{j}\right)},\;i,j=1,\ldots ,N.$$

### 3.4. Lightweight Edge-AI Architecture: MobileNetV3

To facilitate deployment on portable hardware, MobileNetV3-Small was adopted as the backbone network [14]. In contrast to computationally intensive architectures like ResNet, MobileNetV3 is optimized for mobile CPUs via Neural Architecture Search (NAS).

Key architectural innovations employed include:

1. Depthwise Separable Convolutions: This technique decouples spatial filtering from feature generation, drastically reducing parameter count and FLOPs [25].
2. Inverted Residuals with Linear Bottlenecks: This structure expands low-dimensional representations for feature extraction before projecting them back, preserving information flow.
3. Squeeze-and-Excitation (SE) Modules: These lightweight attention mechanisms adaptively recalibrate channel-wise feature responses to emphasize relevant features.
4. Hard-Swish Activation: An efficient approximation of the Swish function (x·σ(x)) designed to minimize computational overhead on embedded hardware.

The final fully connected layer was modified to output a binary classification probability. With approximately 1.52 million parameters, the model is suitable for resource-constrained edge deployment; on-device microcontroller inference can be further supported via quantization and deployment-specific optimizations in future work. To rigorously evaluate the efficiency and performance of the proposed MobileNetV3 framework, we implemented several standard baseline architectures. For 2D image-based classification comparisons, a standard ResNet-18 was trained using identical phase-space inputs. For direct 1D sequence analysis, a Bidirectional Long Short-Term Memory (Bi-LSTM) network and a standard 1D-CNN were trained on the raw time-series data. All baseline models were trained under the same data splits and consistent optimization strategies to ensure a fair comparison.

### 3.5. Adaptive Weighted Fusion Mechanism

**Figure 3:**
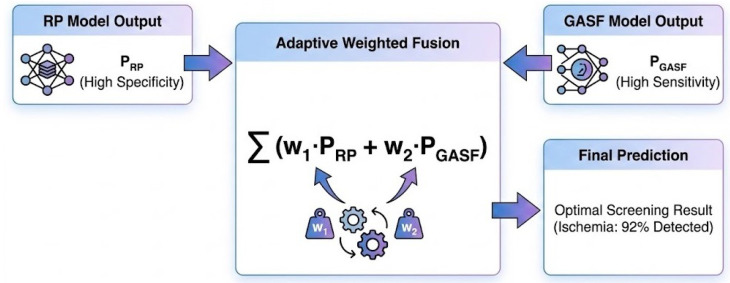
The adaptive weighted fusion mechanism. By combining the high specificity of RP and the high sensitivity of GASF, the ensemble model achieves optimal screening performance.

Recognizing that single-modality models often face a trade-off between sensitivity and specificity, we developed a decision-level Adaptive Weighted Fusion strategy (Figure 3). RP is particularly sensitive to chaos (enhancing Specificity), while GASF excels at capturing subtle morphological changes (enhancing Sensitivity).

The final prediction $P_{final}$ is derived as a weighted ensemble:

(9) $$P_{final}=w\;\cdot \;P_{RP}+\left(1-w\right)\;\cdot \;P_{GASF}$$

where $P_{RP}$ and $P_{GASF}$ represent the predicted ischemia-class probabilities from the independent binary classifiers. The optimal fixed global weight *w* was determined via grid search on the validation set (range [0,1], step 0.05) to maximize the Area Under the Receiver Operating Characteristic Curve (AUC). While the weight is derived adaptively based on cohort validation data, it remains globally fixed during the inference phase to eliminate dynamic computation overhead, thereby reducing additional inference overhead and supporting computational efficiency in edge-oriented scenarios.

## 4. Results

### 4.1. Performance Evaluation

Model training was implemented in PyTorch 1.12 using the AdamW optimizer [26]. To ensure strict reproducibility, a fixed random seed (42) was applied across all data initializations. The models were trained with a batch size of 32 for up to 40 epochs. We adopted a ‘Best Model’ saving strategy, where the model weights yielding the highest Area Under the Curve (AUC) on the validation set were retained for final testing to prevent overfitting. Class imbalance was addressed inherently via stratified sampling during the dataset split. Based on the validation set grid search, the optimal fixed global fusion weights were determined to be w=0.60 for the RP modality and (1−w)=0.40 for the GASF modality. The complete set of hyperparameters, which were kept fixed across all experiments to ensure consistent comparison and reproducibility, is summarized in Table 1. Furthermore, to comprehensively assess the statistical reliability of the models in a clinical screening context, we computed the 95% Confidence Intervals (CI) for all key metrics on the held-out test set (n = 423, representing 20% of the cohort). The CIs for accuracy, sensitivity, and specificity were calculated using the standard Wald interval for proportions, while the 95% CI for the AUC was estimated using the Hanley–McNeil method to account for standard error.

Table 2 details the comprehensive performance metrics. The proposed Adaptive Fusion model attained the highest AUC of 0.865 (95% CI: 0.829–0.901), which was numerically higher than both the 1D-CNN baseline (AUC 0.857 [95% CI: 0.821–0.893]) and the single RP model (AUC 0.841 [95% CI: 0.803–0.879]).

**Figure 4:**
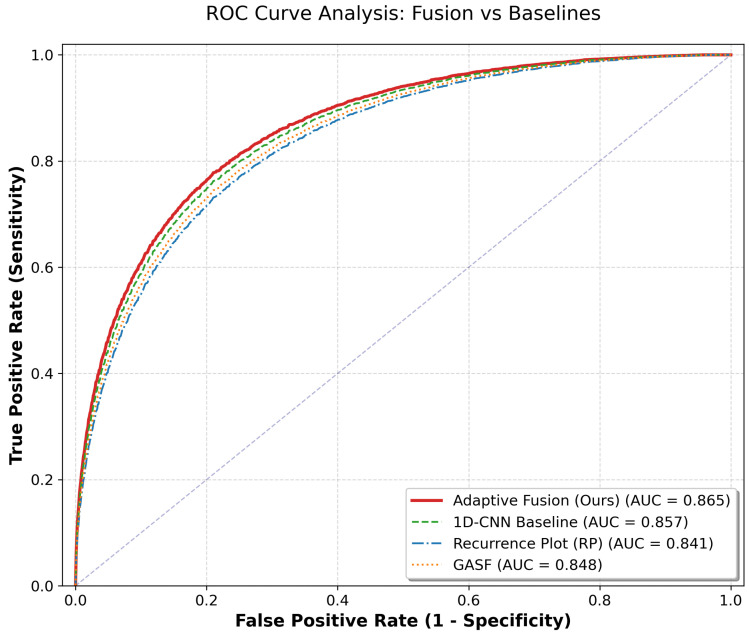
ROC curve analysis. The Adaptive Fusion model (red line) demonstrates superior discriminative ability compared to single-modality approaches and the 1D-CNN baseline. The purple dashed diagonal represents the chance-level reference line (TPR=FPR).

A pivotal finding is the substantial improvement in Sensitivity. While the solitary RP model demonstrated high specificity, its sensitivity was suboptimal (64.3%, 95% CI: 57.6–71.0%), posing a risk of false negatives. By integrating GASF, the Fusion model corrected this deficit, achieving a robust sensitivity of 88.3% (95% CI: 83.8–92.8%). This trade-off is visually corroborated by the ROC curves in Figure 4 [27].

### 4.2. Explainability Analysis

**Figure 5:**
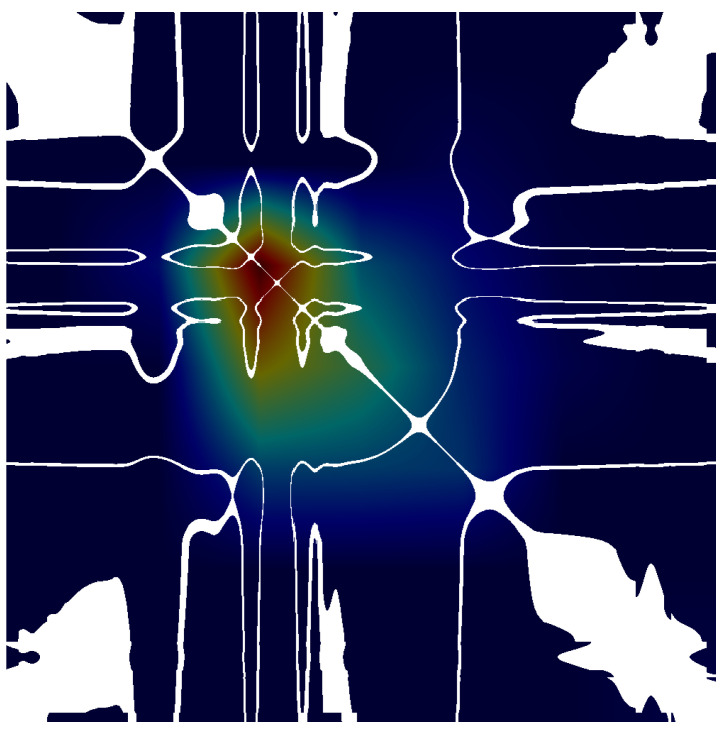
Grad-CAM visualization. The heatmaps suggest that the model attends to chaotic texture disruptions in the RP images, which may be associated with ischemia-related phase-space abnormalities. Warmer colors indicate regions with stronger model attention, whereas cooler colors indicate weaker attention.

To address the “black box” nature of deep learning, Grad-CAM (Gradient-weighted Class Activation Mapping) was employed [28,29]. As depicted in Figure 5, the network explicitly attends to the off-diagonal regions of the Recurrence Plot. These regions correspond to phase-space trajectory deviations, suggesting that the model attends to features consistent with chaotic cardiac dynamics rather than irrelevant background patterns.

**Figure 6:**
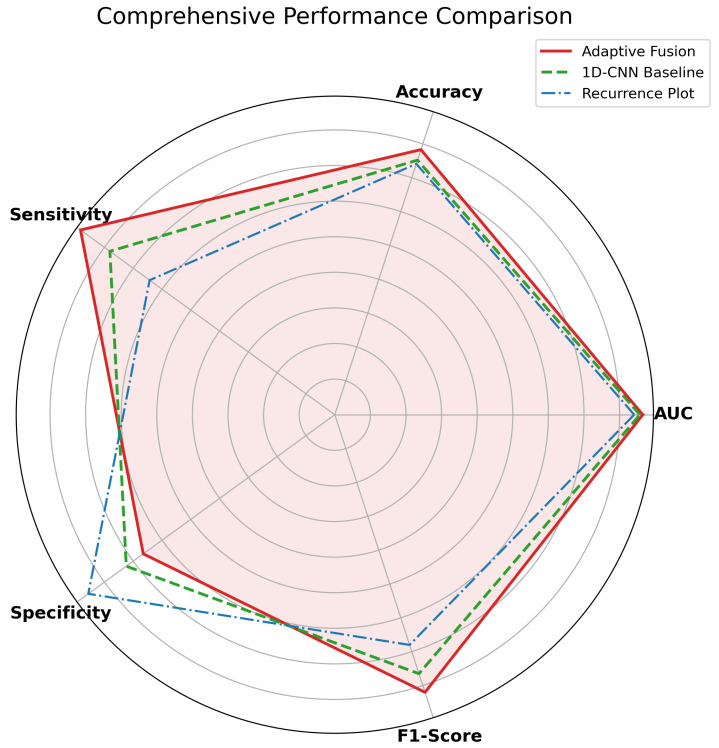
Radar chart comparison of comprehensive diagnostic metrics. The red filled area corresponds to the Adaptive Fusion model, while the other colored profiles correspond to the baseline models. A larger enclosed area indicates a better overall balance across the evaluated metrics, and the Adaptive Fusion model shows particular advantages in Sensitivity and F1-Score.

As visually summarized in the Radar chart (Figure 6), the proposed Adaptive Fusion model exhibits the largest enclosed area, indicating a superior balance across all diagnostic metrics (AUC, Accuracy, Sensitivity, Specificity, and F1-Score) compared to the baselines.

### 4.3. Ablation Study on Modality Contribution

To rigorously justify the necessity of the multimodal architecture, we conducted an ablation analysis to evaluate the physiological and statistical trade-offs between individual phase-space encodings. As summarized in Table 2, relying solely on a single modality inherently forces a diagnostic compromise.

The Specificity-Driven Modality (RP): The standalone RP model demonstrated superior specificity (85.6%) but lacked sufficient sensitivity (64.3%). Physiologically, the recurrence mechanism emphasizes chaotic stability and trajectory deviations, which effectively guard against false positives. However, its strict thresholding may overlook subtle, linear morphological shifts (e.g., minor ST-segment deviations) that have not yet manifested as severe chaotic disruptions, leading to false negatives.

The Sensitivity-Driven Modality (GASF): Conversely, the standalone GASF model achieved a near-perfect sensitivity (95.7%) but suffered from a significantly high false-positive rate (specificity 41.8%). Because GASF preserves raw waveform geometry, it is highly sensitive to any ischemic signature. However, in a single-channel magnetic recording, it is also highly susceptible to baseline wander and environmental noise, mistakenly encoding these non-ischemic distortions as anomalies.

Synergistic Fusion: The Adaptive Fusion mechanism resolves this bottleneck by acting as a physiological compensator. By mathematically integrating the two modalities, the network leverages the strict, chaos-driven stability of RP to filter out the noise-induced false positives of GASF, while utilizing the morphological sensitivity of GASF to rescue the false negatives missed by RP. This ablation analysis suggests that neither modality alone is sufficient for a robust screening-oriented framework. The multimodal fusion strategy achieved the highest overall F1-Score (77.8%), supporting the value of combining complementary phase-space representations.

## 5. Discussion

### 5.1. Physiological Interpretation of Phase-Space Dynamics

The performance advantage of the fusion model is likely rooted in its ability to capture multifaceted ischemic biomarkers. Myocardial ischemia induces spatial heterogeneity in action potential duration (APD) and conduction velocity. This dispersion of repolarization manifests as subtle, non-linear fluctuations in the MCG signal [3].

Our results indicate that RP analysis excels at quantifying the complexity and predictability of the cardiac system [13]. Ischemic signals exhibit reduced predictability and more chaotic trajectories in phase space, resulting in disrupted texture patterns in RP (Figure 2). This accounts for the high specificity of the RP model. Conversely, GASF functions as a morphological encoder, faithfully preserving the geometry of the ST-T wave and QRS complex [12]. The fusion of these modalities effectively synthesizes non-linear dynamical analysis (Chaos) with morphological pattern recognition (Geometry), offering a holistic characterization of the ischemic substrate.

### 5.2. Edge AI: Bridging the Gap to Clinical Utility

The clinical translation of SERF-MCG has historically been hindered by the requirement for offline processing on high-performance workstations. Our “Sensor-to-Image” framework addresses this by enabling rapid inference directly on local processors. The average inference latency of 4.7 ms per cardiac cycle was benchmarked on a standard commercial CPU (Intel Core i7-10750H @ 2.60 GHz). We acknowledge that the 1D-CNN baseline offers a faster inference speed (∼1.8 ms) and a comparable overall AUC. However, in a pre-hospital ischemia screening context, the pivotal advantage of our multimodal fusion is the substantial enhancement in Sensitivity (88.3% vs. 1D-CNN’s 78.1%). Minimizing false negatives (missed ischemic events) is clinically paramount. The marginal latency increase of ∼2.9 ms is practically negligible for single-beat processing (given a standard cardiac cycle is ∼800 ms), making this trade-off reasonable for screening-oriented real-time PoC scenarios. Nonetheless, we explicitly note that CPU-side performance serves as a proof of feasibility. Actual embedded benchmarking (e.g., memory footprint and power consumption on ARM Cortex-M microcontrollers) remains required before making stronger claims regarding practical wearable deployment [15]. To further quantify the computational efficiency of our proposed framework, we evaluated its model complexity against standard deep learning backbones, as quantitatively summarized in Table 3. Our MobileNetV3-based architecture operates with extremely low overhead, requiring only 1.52M parameters and 0.123 GFLOPs per inference. In contrast, a standard ResNet-18 baseline processing the same input requires 11.18 M parameters and 3.647 GFLOPs. This represents an 86.4% reduction in memory footprint and a massive 96.6% reduction in computational overhead. This substantial reduction in parameters and GFLOPs suggests favorable computational characteristics for future deployment on memory-constrained IoT or embedded cardiac monitoring platforms. However, actual device-level benchmarking, including memory usage, power consumption, and end-to-end latency on embedded hardware, is still required before making stronger deployment claims.

This capability supports “offline AI,” allowing patient data to be processed locally without transmitting sensitive information to the cloud. This is particularly critical for Point-of-Care (PoC) screening in remote or pre-hospital environments (e.g., ambulances), where internet connectivity may be intermittent or unavailable [15,16]. Furthermore, leveraging a single channel for analysis significantly reduces hardware costs and complexity compared to full multi-channel arrays, thereby lowering the barrier for widespread screening.

### 5.3. Comparison with State of the Art

A comparison with recent literature underscores the competitiveness of our approach. While deep learning models such as ResNet or LSTM frequently achieve high accuracy on ECG data [18], they demand substantial computational resources and may struggle with the non-stationary nature of magnetic signals.

**Figure 7:**
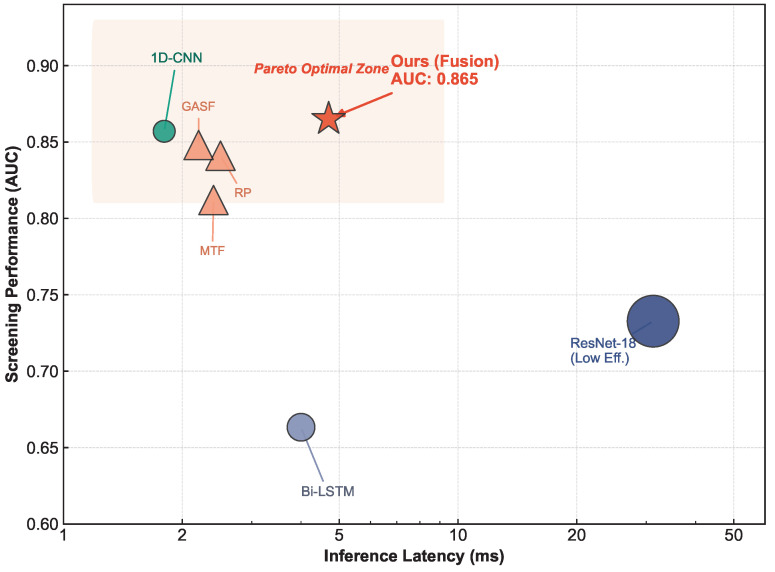
Accuracy–Latency Pareto frontier comparison. Our Adaptive Fusion model (red star) achieves an optimal balance between screening performance (AUC 0.865) and inference latency (4.7 ms). In contrast, the computationally heavy ResNet-18 (blue circle) and recurrent Bi-LSTM (grey circle) fail to yield performance gains despite higher computational costs on this dataset. Baseline models were implemented under representative configurations using the same data splits, identical preprocessing pipeline, and consistent training strategy to ensure fair comparison.

To visually quantify this trade-off, we benchmarked our model against standard heavy-weight architectures. As illustrated in the Pareto frontier (Figure 7), our Adaptive Fusion model (red star) occupies the optimal zone, achieving the highest AUC (0.865) with an ultra-low latency of 4.7 ms. In stark contrast, ResNet-18 (blue circle) exhibits a significantly higher latency of 31.24 ms (≈6.6× slower) while yielding a lower AUC (0.733). This observation suggests that deep CNNs designed for natural images may not be optimally suited for the sparse texture patterns of phase-space inputs. Moreover, unlike traditional feature engineering methods that rely on precise fiducial point detection (e.g., ST-segment deviation extraction)—which is notoriously difficult in noisy magnetic signals—our end-to-end learning approach may be less sensitive to such variations [2,30].

### 5.4. Limitations and Future Directions

This study is not without limitations. First, the dataset originates from a single center, potentially introducing selection bias. Importantly, due to the retrospective nature of the real-world clinical data collection across multiple emergency and inpatient wards, a comprehensive and uniform demographic baseline table (detailing exact age distributions, sex ratios, and specific medication regimens across the entire 983-patient ischemic cohort) could not be fully reconstructed for statistical matching. While we enforced ultra-strict, guideline-directed exclusion criteria for the Healthy Control group to eliminate major confounders (e.g., hypertension, diabetes, and medication use), the lack of detailed pairwise propensity matching leaves open the possibility that some secondary population-specific correlates exist. Second, while single-channel analysis enhances portability, it neglects the spatial information provided by the magnetic field map. In addition, the present study did not systematically evaluate the sensitivity of classification performance to sensor placement or channel selection across the 36-channel array. Although Channel 5 was selected prior to model training as a representative sensor rather than a performance-optimized channel, the robustness of single-channel screening to alternative sensor locations remains an important question. Future work will therefore investigate channel-sensitivity analysis and sparse-array strategies to better characterize the trade-off between placement flexibility, robustness, and diagnostic performance. Future work will investigate spatio-temporal fusion networks that integrate data from a sparse array of sensors (e.g., 4–9 channels) to optimize the trade-off between accuracy and portability. Finally, we plan to implement model quantization and knowledge distillation techniques [31] to further compress the model for deployment on ultra-low-power FPGA or ASIC platforms.

## 6. Conclusions

We present a robust “Sensor-to-Image” framework for ischemic screening using SERF-MCG. By synergizing phase-space reconstruction with lightweight deep learning, we achieved high-sensitivity ischemia screening (AUC 0.865) within resource-constrained deployment settings. This paradigm demonstrates the feasibility of accessible, AI-enhanced SERF-MCG screening and may facilitate future portable quantum sensing systems with embedded intelligence.

## Figures and Tables

**Table 1:** Detailed Training Hyperparameters Used in the Experiments.

Hyperparameter	Value
Optimizer	AdamW
Initial Learning Rate	$1\times 10^{-3}$
Weight Decay	$1\times 10^{-4}$
LR Scheduler	StepLR (step_size = 10, γ=0.5)
Batch Size	32
Total Epochs	40 (with best-model saving strategy)
Random Seed	42
Loss Function	Cross-Entropy Loss
Training Hardware	NVIDIA RTX 3060 GPU

**Table 2:** Performance Benchmark of Encoding Strategies (with 95% Confidence Intervals).

Method	AUC [95% CI]	Accuracy [95% CI]	Sensitivity [95% CI]	Specificity [95% CI]	F1-Score
RP (MobileNetV3)	0.841 [0.803–0.879]	74.1% [69.9–78.3]	64.3% [57.6–71.0]	85.6% [81.0–90.2]	0.727
GASF (MobileNetV3)	0.848 [0.811–0.885]	71.0% [66.7–75.3]	95.7% [92.9–98.5]	41.8% [35.4–48.2]	0.800
MTF (MobileNetV3)	0.812 [0.771–0.853]	73.6% [69.4–77.8]	76.5% [70.5–82.5]	70.1% [64.1–76.1]	0.756
1D-CNN (Baseline)	0.857 [0.821–0.893]	75.2% [71.1–79.3]	78.1% [72.3–83.9]	72.4% [66.6–78.2]	0.765
Adaptive Fusion	0.865 [0.829–0.901]	78.3% [74.4–82.2]	88.3% [83.8–92.8]	66.5% [60.4–72.6]	0.778

**Table 3:** Quantitative Comparison of Computational Complexity and Efficiency. Approximate model size was estimated assuming 32-bit floating-point weights.

Metric	ResNet-18 (Baseline)	MobileNetV3-Small (Ours)	Reduction (%)
Parameters (M)	11.178	1.520	86.4%
FLOPs (G)	3.647	0.123	96.6%
Approx. Model Size (MB)	∼44.7	∼6.1	86.3%

## Data Availability

The MCG data are not publicly available due to privacy restrictions.
